# From Skin Deep to Life-Threatening: A Case of Necrotizing Fasciitis Masquerading as Ecchymosis

**DOI:** 10.7759/cureus.87125

**Published:** 2025-07-01

**Authors:** Abigail Alorda, Sadman Chowdhury, Stephanie Cohen, Jovans Lorquet, Shayne Gue

**Affiliations:** 1 Emergency Medicine, HCA Florida Osceola Hospital, Kissimmee, USA; 2 Emergency Medicine, University of Central Florida College of Medicine, Orlando, USA; 3 Medical Education, University of Central Florida College of Medicine, Orlando, USA; 4 Emergency Medicine, BayCare Health System/St. Joseph's Hospital, Tampa, USA

**Keywords:** diabetic wound, lrinec score, necrotizing fasciitis, necrotizing skin and soft tissue infections, sepsis, staphylococcus-associated glomerulonephritis

## Abstract

Necrotizing fasciitis (NF) is a rare but life-threatening infection characterized by rapid and extensive tissue necrosis. Early identification is often difficult, particularly when physical exam findings are subtle or atypical. We present the case of a man in his forties with poorly controlled type 2 diabetes mellitus who arrived at the emergency department after noticing discoloration and swelling of his right great toe. The patient had minimal pain but was noted to be hypotensive and tachycardic on arrival. Laboratory workup revealed hyperglycemia, hyponatremia, and elevated lactic acid. Imaging was negative for fracture or subcutaneous air. Due to the rapid progression of symptoms and abnormal vital signs, general surgery was consulted to evaluate for NF. The patient was admitted and subsequently developed a temperature of 106°F, requiring escalation of care. He underwent surgical debridement and amputation of the right hallux. Wound and blood cultures grew *Staphylococcus aureus*, and he was later diagnosed with Staphylococcus-associated glomerulonephritis, confirmed by renal biopsy. This case demonstrates the importance of early recognition and intervention in NF, particularly in patients with diabetic neuropathy, and highlights a rare complication of *S. aureus* infection.

## Introduction

Necrotizing fasciitis (NF) is a severe, life-threatening infection that is characterized by rapid and extensive necrosis of the muscle fascia and subcutaneous tissue [1-4]. It is a rare condition in the United States but is usually associated with significant complications [4,5]. The infection usually spreads quickly along the fascial plane without a significant initial change of overlying skin, potentially delaying diagnosis and intervention [4,6,7]. Due to its aggressive nature and often delayed diagnosis, it is associated with high morbidity and mortality [2,3,5]. This case report presents a clinical description of a patient who presented with a near typical case of NF but ended up with a rare complication of Staphylococcus-associated glomerulonephritis (SAGN).

## Case presentation

Patient information

An adult male patient, in his forties, presented to the emergency department (ED) with a chief complaint of discoloration of his toe. The patient reported that he had been working the day prior at his construction job when a brick had fallen onto his boot-covered foot. When he was showering that following morning, he noted that the area seemed to have changed in color and become more swollen. The patient denied any significant pain at that time but did report he had previously diagnosed diabetic neuropathy. In addition to the discoloration and swelling of the toe, the patient reported subjective fever and chills at home prior to arrival.

**Figure 1:**
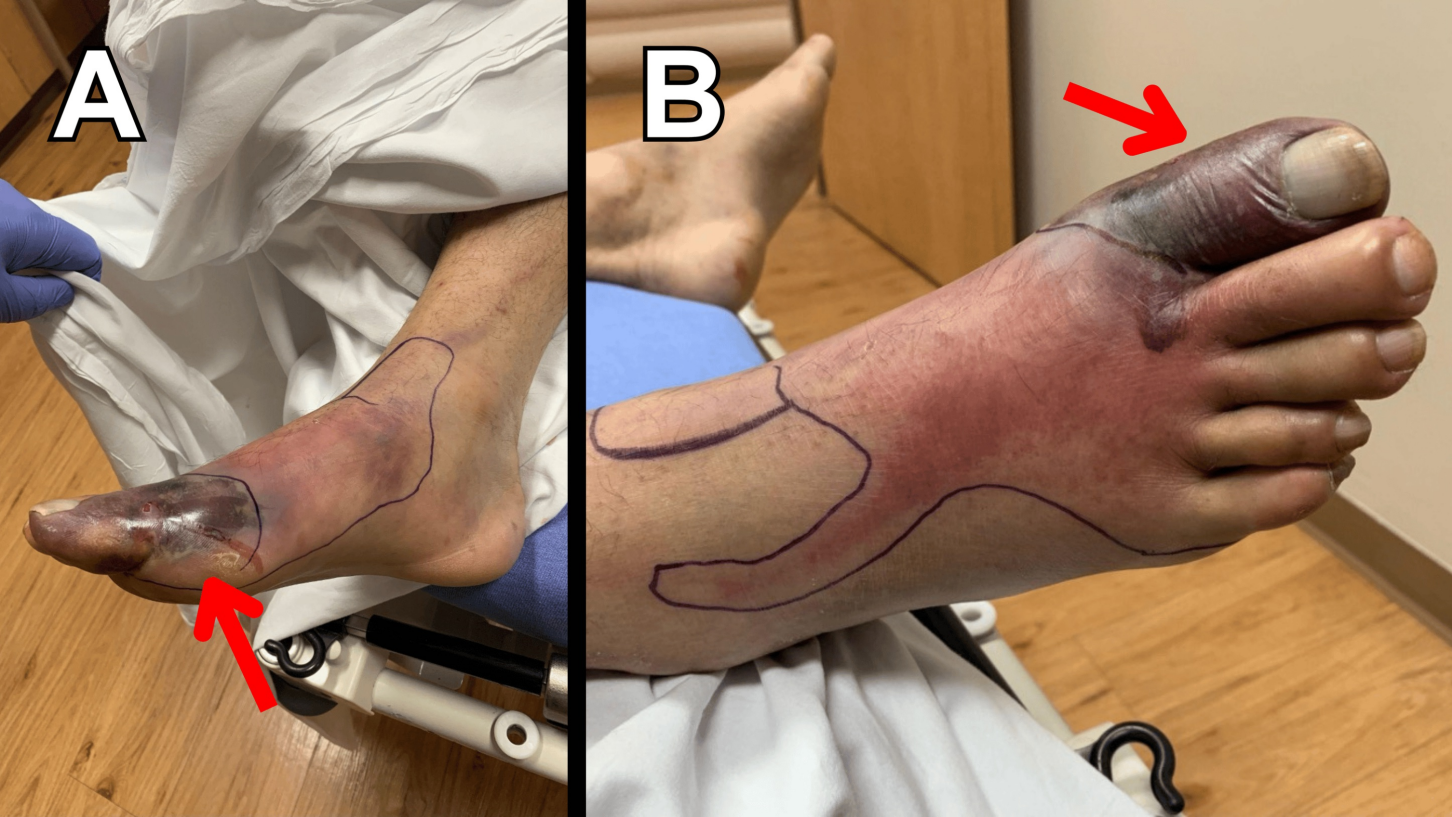
Medial (A) and anterior (B) views of ecchymosis (red arrow) to the dorsal aspect of the right great toe with surrounding erythema.

Upon arrival to the ED, although the patient was noted to be afebrile (98.9 degrees F), he was found to be tachycardic (112 beats per minute) and hypotensive (95/58 mmHg). A “sepsis alert” was initiated at our facility, which includes prioritizing the collection and analysis of blood cultures and the serum lactic acid level, along with the timely administration of broad-spectrum antibiotics and an intravenous fluid bolus. His physical examination revealed an ecchymotic area to the dorsal aspect of the right great toe with surrounding erythema; there was no tenderness to palpation or crepitus identified, and his extremity was otherwise neurovascularly intact (see Figure 1). Furthermore, there was no temperature discrepancy compared to the contralateral extremity, nor was proximal lymphadenopathy noted. Based on this presentation, initial differential considerations included cellulitis, gout, deep vein thrombosis, or necrotizing skin, and soft tissue infection.

Diagnostic assessment

**Figure 2:**
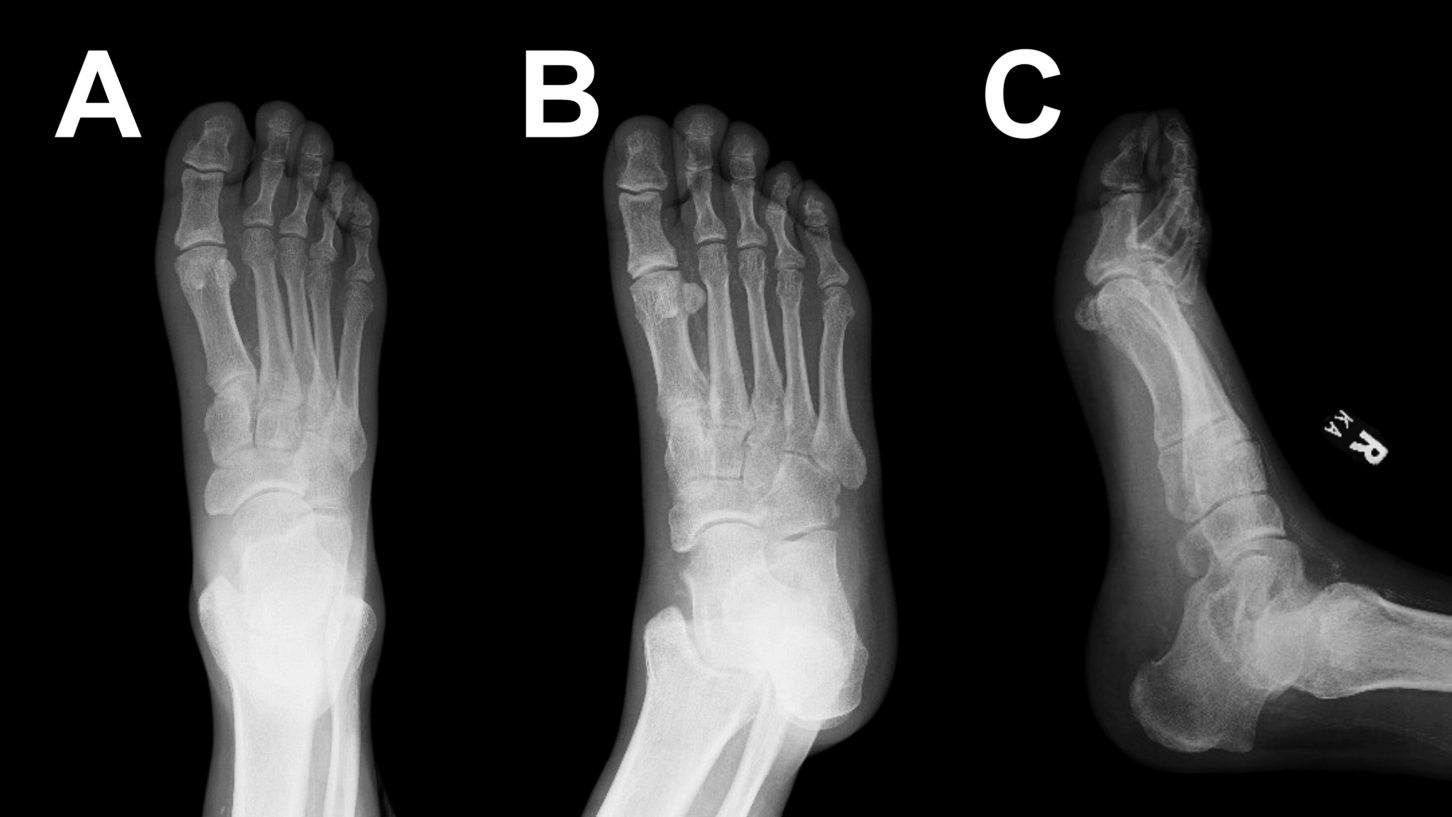
Anteroposterior (A), oblique (B), and lateral (C) plain radiographic imaging of the foot negative for acute fracture or subcutaneous air.

Based on the initial differential described, plain radiography and diagnostic laboratory studies were ordered. Initial radiographic imaging with X-ray was negative for acute fracture or subcutaneous air (see Figure 2). Laboratory studies were significant for normal white blood cell count (9800 cells/microliter), anemia (hemoglobin of 12.2 g/dL), hyponatremia (sodium of 125 mmol/L), hyperglycemia (glucose of 485 mg/dL), elevated lactic acid (3.1 mmol/L), and elevated BUN (34 mg/dL) and creatinine (1.9 mg/dL), with no baseline available for comparison. Additionally, his coagulation panel was abnormal with a prothrombin time (PT) of 16.7 seconds and International Normalized Ratio (INR) of 1.4. See Table 1 for complete laboratory results. Due to the patient's vital signs and physical exam abnormalities, as well as the rapid progression of his symptoms, the general surgery service was consulted emergently to assess for NF.

**Table 1:** Laboratory results at initial presentation in the emergency department.

Laboratory test	Patient value	Normal range	Units
White blood cell (WBC) count	9.8	4.0-10.5	x10^3^ cells/µL
Hemoglobin (Hgb)	12.2	13.7-17.5	g/dL
Hematocrit (Hct)	33.1	40.1-51.0	%
Platelet count	145	150-400	x10^3^ cells/µL
Sodium	125	136-145	mmol/L
Potassium	3.6	3.7-5.1	mmol/L
Chloride	95	98-107	mmol/L
Carbon dioxide	20	21-32	mmol/L
Blood urea nitrogen (BUN)	34	6-24	mg/dL
Creatinine	1.9	0.55-1.3	mg/dL
Glucose	485	74-106	mg/dL
Lactic acid	3.1	0.4-2.0	mmol/L
Prothrombin time (PT)	16.7	10.0-12.8	seconds
International normalized ratio (INR)	1.4	0.8-1.1	

Therapeutic intervention and outcome

While awaiting general surgery consultation, the patient received intravenous cefepime (2 grams) and vancomycin (1 gram) for broad-spectrum antibiotic coverage in the setting of potential sepsis. Additionally, insulin was administered due to profound hyperglycemia (485 mg/dL). When the suspicion for NF became more likely, clindamycin (600 mg) and metronidazole (500 mg) were added to the antibiotic regimen. The patient was admitted to the progressive care unit for further evaluation and management.

Thirty minutes after admission, the patient was found to be febrile with a temperature of 106° F. An indwelling Foley catheter with a temperature probe was placed for continued monitoring and accuracy. The patient was given acetaminophen and ice packs were placed for hyperthermia. The general surgery team was made aware of this acute change and upgraded the patient to intensive care unit-level care.

On hospital day 2, the patient underwent surgical debridement and right hallux amputation with partial resection of the first metatarsal in the affected area. His blood culture was positive for *Staphylococcus aureus*, and he later developed acute renal failure from SAGN confirmed on renal biopsy. The patient was found to have poorly controlled diabetes with a hemoglobin A1c of 11 mg/dL.

The patient was discharged on hospital day 13 to a rehabilitation facility with a temporary dialysis catheter in place, and follow-up plans with nephrology were arranged. Renal recovery is ongoing, with the patient requiring intermittent dialysis at the time of discharge.

## Discussion

NF is part of a subset of severe, life-threatening soft-tissue infections commonly referred to as necrotizing skin and soft-tissue infections (NSTIs). These terms are often used interchangeably in the literature. NSTIs are usually defined as infections of any layer(s) within the soft tissue compartment including dermis, subcutaneous tissue, superficial fascia, deep fascia, and muscle [1]. Its aggressive nature and mechanism of spread create a potential delay in diagnosis and intervention, leading to high morbidity and mortality.

As seen in this case, the overwhelming majority of the time, a break in the skin barrier allows bacteria to invade the subcutaneous tissue and fascial plane. Comorbidities such as diabetes mellitus, peripheral vascular disease, alcoholism with liver disease, and cancer with immunosuppression can predispose to NF, with diabetes being the most significant among them [2]. Our patient presented with severely uncontrolled diabetes with a hemoglobin A1c of 11 mg/dL, predisposing him to NF.

NF is predominantly a bacterial infection, with gram-positive cocci such as *S. aureus* and Streptococci being the main culprits. It is also important to note that polymicrobial infection can also occur with a mixture of gram-negative and anaerobic bacterial involvement [3]. Although rare, with an annual incidence of 0.4 per 100,000 people in the US, mortality remains between 20-30% [4,5].

Differentiating NF from other common infectious entities, such as cellulitis, is often difficult initially, which may lead to a delay in diagnosis. Some findings that favor NF include erythema with soft-tissue edema, out-of-proportion pain, fever, hemorrhagic bullae, and hypotension [6]. Plain radiograph and computed tomography (CT) of the affected region may demonstrate gas in the soft tissue, with CT having far superior sensitivity (88.5%) and specificity (93.3%) [5]. Although the presumptive diagnosis of NF is primarily a clinical one, imaging and laboratory values may provide useful information if the diagnosis is uncertain [4]. Clinical decision-making tools, such as the Laboratory Risk Indicator for Necrotizing Fasciitis (LRINEC), may help differentiate NF from other soft tissue infections, but existing tools exhibit poor sensitivity and are thus insufficient in ruling out NF [5-8]. In this case, the patient initially presented with erythema and soft tissue edema; he was afebrile and exhibited no tenderness to palpation. This could be due to atypical presentation or his history of uncontrolled diabetes leading to neuropathy. However, due to his unstable vital signs and abnormal laboratory values, general surgery was consulted to rule out NF.

NF can result in many serious complications such as septic shock, multiple organ failure, loss of extremities, severe scarring, and death. In this case, wound and blood cultures were positive for* S. aureus*. One of the rare complications noticed in this case was SAGN. SAGN is a rare type of post-infectious glomerulonephritis. It is characterized by immune complex-mediated glomerulonephritis associated with active *Staphylococcus* infection [9]. The pathogenesis is similar to other post-infectious glomerulonephritis that involves the deposition of immune complexes in the basement membrane of the glomerulus, subsequently causing inflammation and injury [10].

## Conclusions

This case underscores the importance of early recognition and timely intervention in NF, particularly in patients with diabetic neuropathy who may lack classical pain symptoms. Emergency clinicians must maintain a high index of suspicion when systemic signs such as hypotension, hyperglycemia, and lactic acidosis are present, even when physical findings are subtle. While the primary focus of this report is the aggressive course and clinical management of NF, it is also notable that the patient developed a rare immune-mediated renal complication. The recognition of such post-infectious sequelae, such as SAGN, highlights the importance of continued monitoring and multidisciplinary care throughout the patient’s clinical course.
